# Reversible Photoisomerization in Thin Surface Films from Azo-Functionalized Guanosine Derivatives

**DOI:** 10.1021/acsomega.1c01879

**Published:** 2021-06-07

**Authors:** Matjaž Ličen, Stefano Masiero, Silvia Pieraccini, Irena Drevenšek-Olenik

**Affiliations:** † Faculty of Mathematics and Physics, University of Ljubljana, Jadranska 19, SI-1000 Ljubljana, Slovenia; ‡ Dipartimento di Chimica “Giacomo Ciamician”, Alma Mater Studiorum—Università di Bologna, Via San Giacomo 11, I-40126 Bologna, Italy; § Department of Complex Matter, Jožef Stefan Institute, Jamova 39, SI-1000 Ljubljana, Slovenia

## Abstract

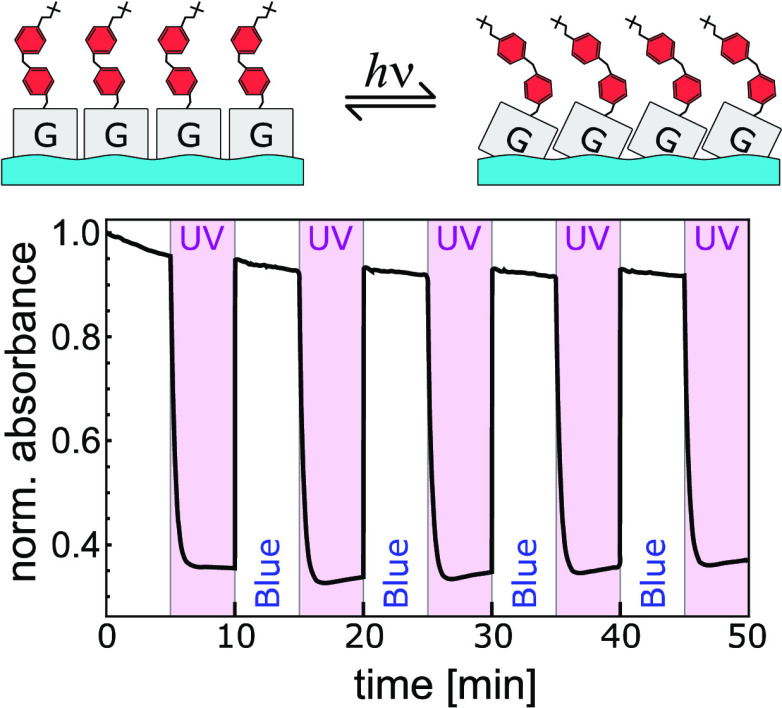

Two novel azo-functionalized guanosine derivatives were synthesized, and their photoisomerization process was investigated in molecular monolayers at the air–water interface and in the Langmuir–Blodgett (LB) films on solid substrates. Measurements of surface pressure vs area isotherms, surface potential measurements, UV–visible (vis) absorption spectroscopy, Brewster angle microscopy (BAM), and atomic force microscopy (AFM) were performed. Despite not having a typical amphiphilic molecular structure, the derivatives formed stable films on the water surface. They could also undergo repeated photoisomerization in all of the investigated thin-film configurations. The observations suggest that in the films at the air–water interface, the molecules first exhibit a conformational change, and then they reorient to an energetically more favored orientation. In the LB films transferred onto solid substrates, the isomerization process occurs on a similar time scale as in solution. However, the isomerization efficiency is about an order of magnitude lower than that in solution. Our results show that DNA nucleobases functionalized with azobenzene moieties are suitable candidates for the fabrication of photoactive two-dimensional (2D) materials that can provide all beneficial functionalities of DNA-based compounds.

## 1 Introduction

Because of their versatility and many appealing structural and physical properties, DNA and its components and their derivatives are emerging candidates for use in a wide range of applications, from memory devices,^1,2^ various self-assembled nanostructures,^3−12^ molecular computing units,^13−21^ and nanosensors^22−27^ to biocompatible electronics.^28−41^ Among the four DNA nucleobases, guanosine stands out due to its ability to form stable and robust noncanonical structures with other guanosine molecules, in particular G-quadruplexes. Due to this ability, this nucleobase has recently become a target of extensive investigations for use in nanomaterials^7,42−49^ and DNA-targeting drugs.^50−52^

Most of the above-mentioned technological applications rely on hydrogen bonding among guanine units. Therefore, the development of effective mechanisms to control this bonding is a prerequisite for further advancing of the field. Among different methods for controlling hydrogen bonding,^53^ light-driven control is often favored because it is noninvasive and offers precise spatial and temporal regulation. Such a control can be implemented by modification of a nucleobase with a photoactive moiety so that its binding to other nucleobases can be regulated by irradiation with light of a specific wavelength.^54−56^ The most common of such moieties are azobenzene derivatives. They change their configuration from a stretched trans to a bent cis isomer when irradiated with light of one wavelength (typically in the UV spectral range) and back from cis to trans isomer when irradiated with light of another wavelength (usually in the visible (vis) spectral range). Alternatively, cis-to-trans back-isomerization can also take place via spontaneous thermal relaxation. Their picosecond switching times^57^ and photochemical stability^58^ make azobenzene derivatives a popular choice for adding photoactive properties to various materials.

Many nanotechnological applications are based on thin-film-type structures. One of the most precise methods to form such structures is the Langmuir–Blodgett (LB) technique. In the LB technique, two-dimensional (2D) materials are fabricated by first assembling the amphiphilic molecules into a compact molecular monolayer at the air–water interface (Langmuir monolayer) and then transferring the obtained film onto a solid substrate. The technique enables the fabrication of large-scale 2D materials^59,60^ suitable for photovoltaics,^61^ molecular electronics,^41,62^ model membranes,^63^ functionalized coatings,^64^ etc.^65^

It is known that base pairing can take place in the Langmuir film of a selected nucleoside when its complementary nucleobase is introduced either to the air–water interface^66−69^ or into the water subphase.^70−80^ In our recent study, we demonstrated that hydrogen bonding between guanosine and its complementary nucleoside (cytidine) at the air–water interface could be manipulated by optical irradiation.^81^ Besides the azobenzene moiety, guanosine molecules used in that study also possessed two hydrocarbon chains, which are generally believed to be necessary for the formation of molecular films at the water surface. In this work, we report on the light-driven control of guanosine derivatives functionalized solely with the azobenzene moieties. The results show that these very simple derivatives also form stable Langmuir and LB films whose configurational state can be repeatedly switched by optical irradiation.

## 2 Experimental Results

### 2.1 Photoisomerization in Solution

**Figure 1:**
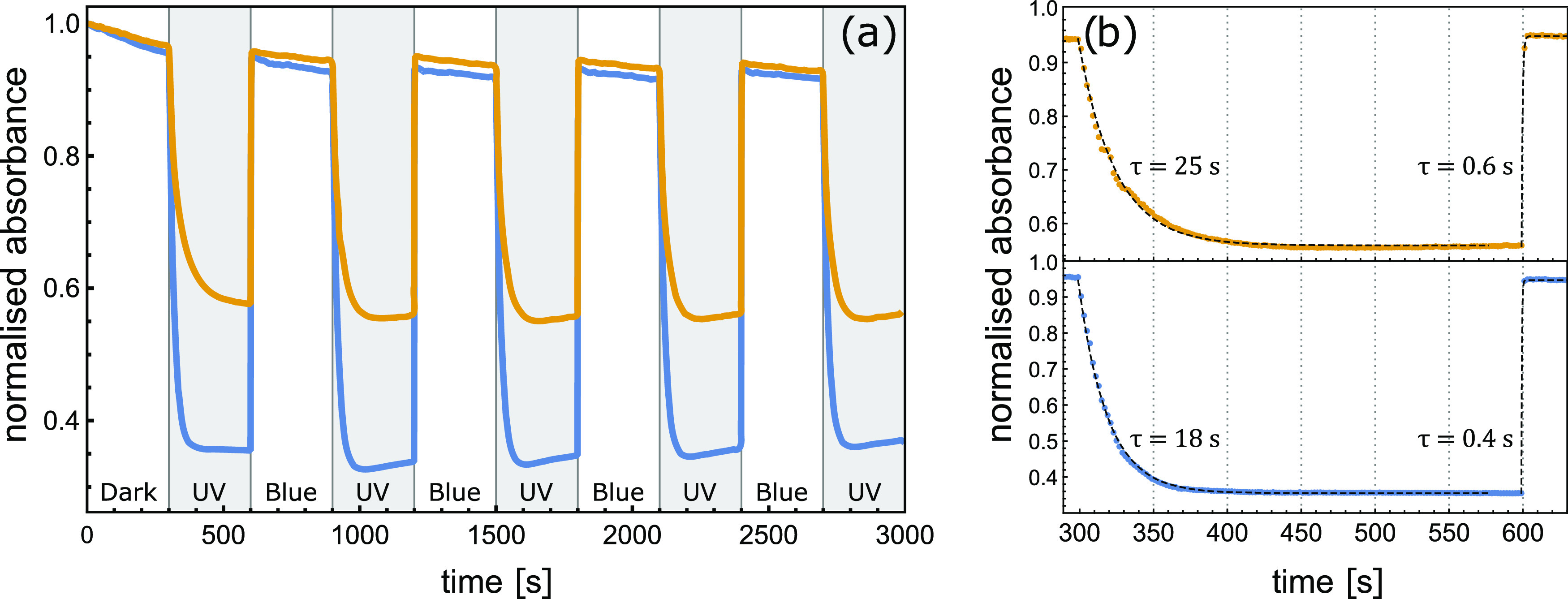
Temporal modifications of normalized absorbance ε(*t*)/ε(*t* = 0) at 330 nm detected in chloroform solutions of GAzo (blue) and GAzo_3_ (yellow) during alternating irradiation with blue and UV light. Intervals of UV irradiation are shaded in gray. The type of irradiation is also marked at the bottom of the plot. Subfigure (b) shows fits of an exponential function (dashed lines) to the data for one cycle of irradiation with UV and blue light. Characteristic times obtained from the fits are denoted next to the curves.

To obtain reference data for the efficiency and speed of photoisomerization processes, we first experimented with 30 μM chloroform solutions of GAzo and GAzo_3_ molecules. The solutions were alternatingly irradiated with UV and blue light for 5 min, causing the molecules to switch from trans to cis configuration and back. The resulting changes in absorbance measured at 330 nm (absorption peak of the trans isomers, see Figure 8 in Section 4) are shown in Figure 1.

When irradiated with UV light, the absorbance of GAzo solution dropped by approximately 60%, while the absorbance of GAzo_3_ solution dropped by 40%. The characteristic times of the transitions, obtained by fitting exponential functions to the experimental data (Figure 1b), are 18 s for the trans–cis transition of GAzo, 25 s for the trans–cis transition of GAzo_3_, 0.4 s for the cis–trans transition of GAzo, and 0.6 s for the cis–trans transition of GAzo_3_. The fact that absorbance does not remain constant when saturation is reached, but gradually increases during continued UV irradiation and decreases during blue irradiation, indicates some irreversible changes (bleaching) caused by the irradiation.

### 2.2 Photoisomerization in Langmuir films

**Figure 2:**
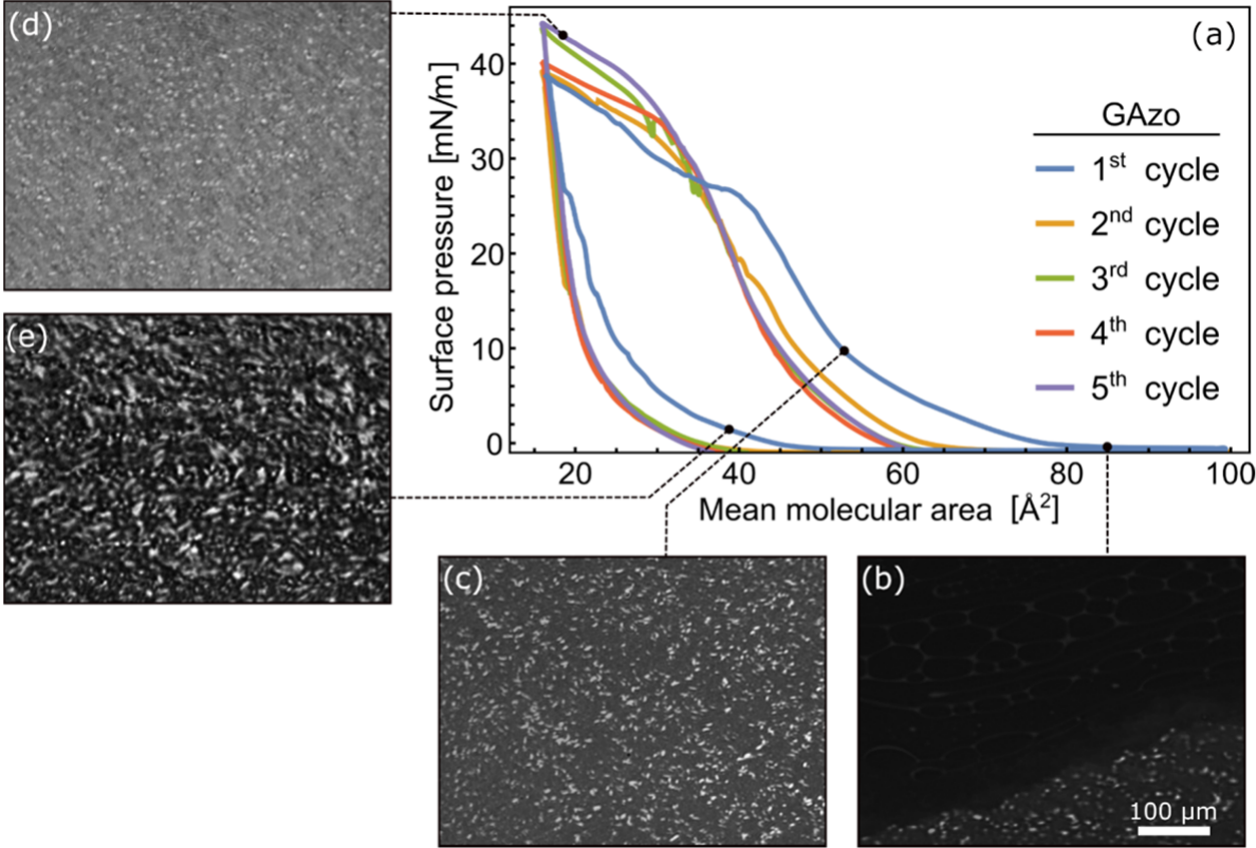
(a) Surface pressure vs mean molecular area isotherms measured during five consecutive compression–expansion cycles of Langmuir films of GAzo molecules. Data measured in different cycles are shown with different colors, as indicated in the legend. Images (b)–(e) are Brewster angle microscopy (BAM) images of the film captured during the first compression–expansion cycle. The scale bar in subfigure (b) represents the scale for all BAM images. The stages in the first compression–expansion cycle in which the images were taken are indicated on the isotherm.

**Figure 3:**
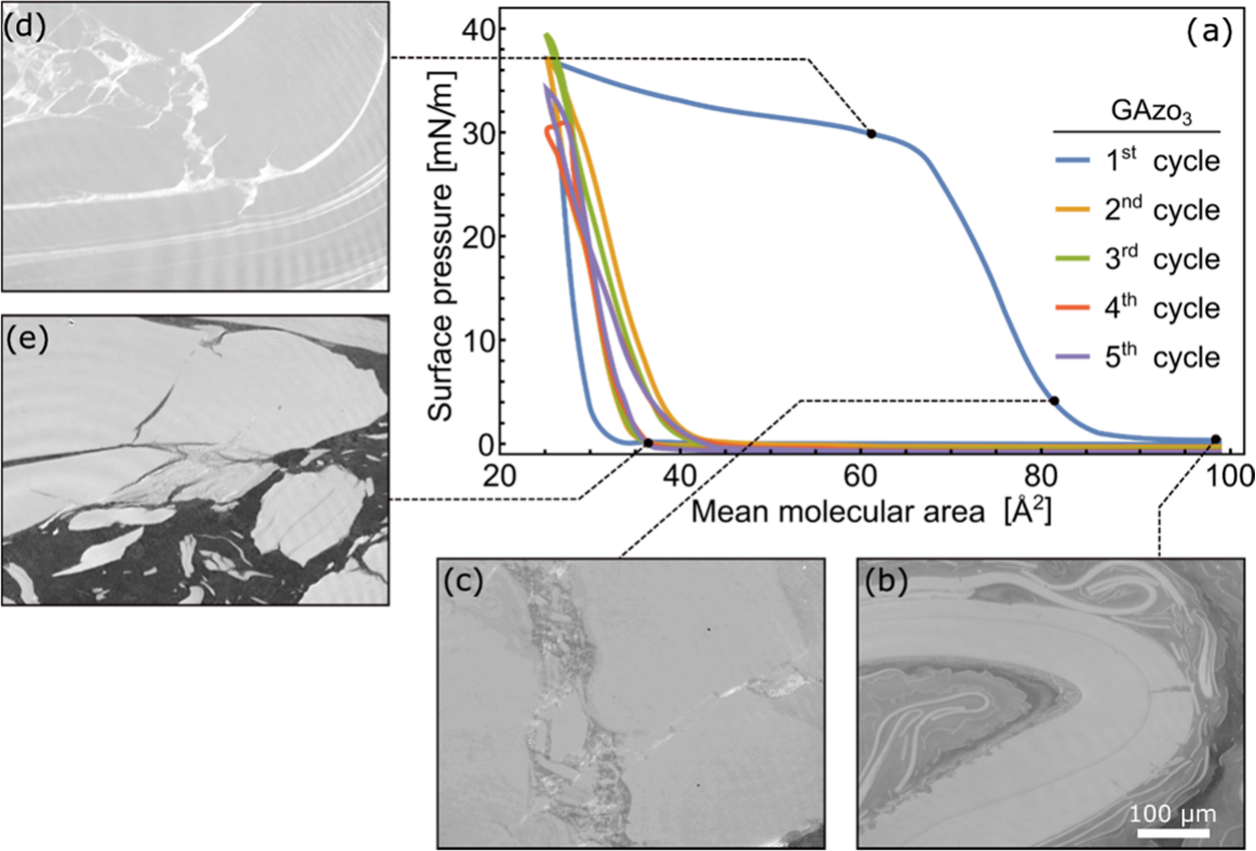
Surface pressure vs mean molecular area isotherms measured during five consecutive compression–expansion cycles of Langmuir films of GAzo_3_ molecules. Data measured in different cycles are shown in different colors, as indicated in the legend. Images (b)–(e) are Brewster angle microscopy images of the film captured during the first compression–expansion cycle. The scale bar in subfigure (b) represents the scale for all BAM images. The stages in the compression–expansion cycle in which the images were taken are indicated on the isotherm.

The surface pressure vs area isotherms measured in five consecutive compression–expansion cycles of the GAzo and GAzo_3_ Langmuir films and the Brewster angle microscopy (BAM) images of typical film morphologies observed at different stages of the first expansion–compression cycle are shown in Figures 2 and 3, respectively.

In the case of GAzo (Figure 2a), the surface pressure starts increasing at a mean molecular area of approximately 80 Å^2^ and briefly plateaus at around 45 Å^2^. With further compression, it keeps increasing until it reaches the maximum value of 44 mN/m. The hysteresis present between the compression and the expansion parts of the cycle indicates binding between the molecules. Such hysteresis appears in Langmuir films of lipophilic nucleoside derivatives of various nucleobases and was investigated in more detail in our previous work.^82^

The corresponding BAM images reveal structures of varied densities that are present on the water surface even before the start of the film compression (Figure 2b). As the film is compressed, the frequency with which such denser structures are observed increases while the surface pressure still remains zero. The starting rise of surface pressure coincides with full coverage of the water surface with the GAzo molecules. The more the film is compressed, the brighter the images are, and some bright specks appear on top of the initial film (Figure 2c). With further compression they become more and more numerous (Figure 2d). No noticeable change in the structure accompanies the resolved change in compressibility. During the initial stages of expansion, the appearance of the film remains the same. Only when the surface pressure gets close to zero again, does the film start to slowly break apart into smaller pieces (Figure 2e).

In the second compression/expansion cycle, the surface pressure starts increasing at a significantly lower mean molecular area than in the first cycle, i.e., at about 65 Å^2^. The plateau is not present anymore, which could indicate some binding between the molecules that is not reversed upon expansion of the film formed during the first compression. However, in all of the subsequent cycles, the isotherms are almost identical to the one observed in the second cycle, indicating that all irreversible changes happen in the first cycle.

During the first compression of GAzo__3__ molecules (Figure 3a), the surface pressure starts increasing at a similar value of the mean molecular area as in the case of GAzo films (∼90 Å^2^), which signifies that the effective surface area of the molecules is dictated predominantly by the size of the guanosine moiety. However, the compressibility of the GAzo__3__film is lower, and the plateau is reached at a molecular area of around 70 Å^2^.

Similar to GAzo films, BAM images of GAzo__3__films reveal visible structures present on the water surface already immediately after the deposition. After some compression of the barriers, but before the surface pressure begins to increase, lamellar structures can be observed (Figure 3b), which indicates some degree of molecular order. Similar lamellar packing was also observed in Langmuir and LB films of other lipophilic guanosine derivatives.^83,84^ It is attributed to the formation of G-ribbon-type assemblies.^85^

When the entire water surface is covered by the lamellar multilayer (Figure 3c), further compression causes surface pressure to increase. From here on, the image observed by BAM is almost static, suggesting that the film is very rigid and that the molecules are locked in place. Surprisingly, this remains the case even after the surface pressure plateaus (Figure 3d). However, it is possible that changes in the film structure causing the plateau occurred near the barriers of the LB trough and not in the center of the film, where the BAM images were taken.

When the GAzo__3__film is expanded, the surface pressure very quickly drops to zero. The drop is accompanied by the appearance of cracks in the film, followed by its eventual breakup (Figure 3e). Remarkably, flakes of the broken film can still be observed even after the film had been completely expanded. In the second cycle, the surface pressure starts increasing at a much lower mean molecular area of 40 Å^2^. BAM images suggest that during the second compression cycle, the flakes that remained on the water surface from the first cycle are simply gathered up and compressed once again, leading to the striking change in the shape of the detected isotherm. Both derivatives, GAzo__3__ and GAzo, exhibit quite nonclassical surface pressure vs area dependence. We assume that the investigated part of the isotherms corresponds to the liquid-expanded phase.

**Figure 4:**
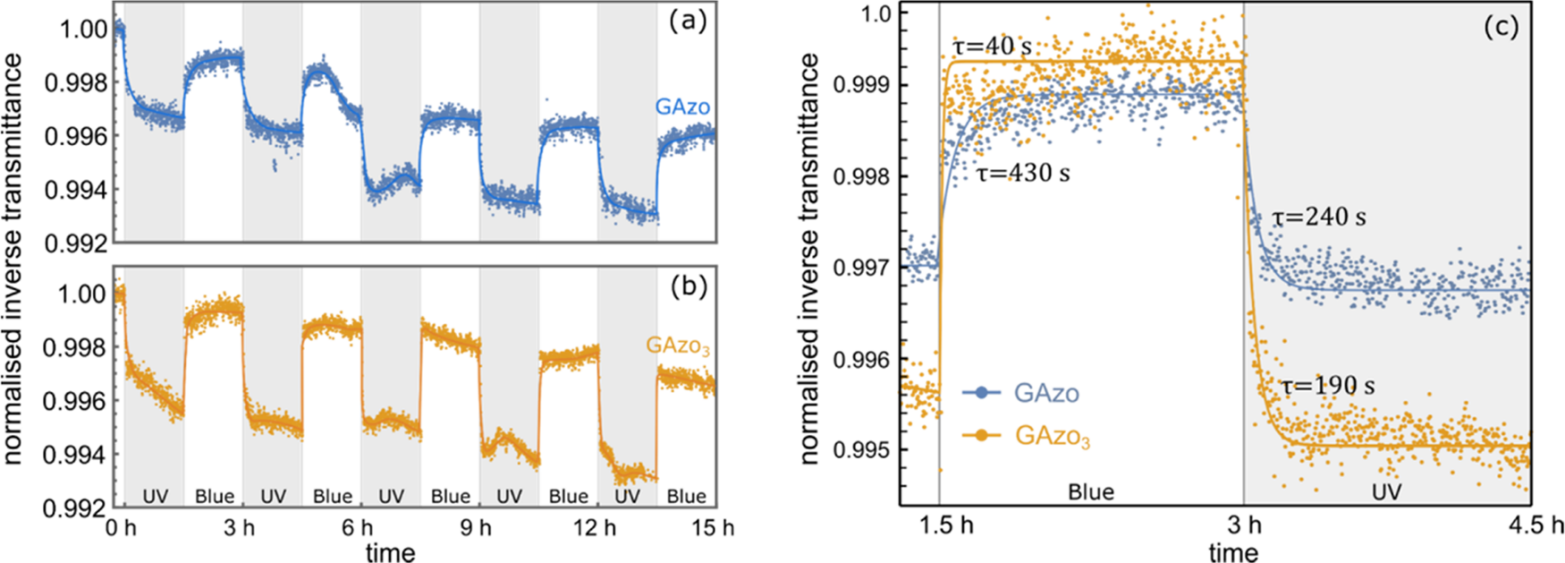
Changes of the inverse transmittivity of the Langmuir films of GAzo (a) and GAzo_3_ (b) during subsequent irradiation with UV and blue light. Intervals of UV irradiation are shaded in gray. The type of irradiation is also marked at the bottom of the plot. Fits of exponential functions to the data for one characteristic interval are shown in subfigure (c). Characteristic times obtained from these fits are denoted next to the curves.

After characterizing the compression/expansion isotherms of the films, we focused on the effect of optical irradiation on their UV–vis transmission and absorption properties. The observed changes in the optical transmission of the Langmuir films of GAzo and GAzo_3_ during subsequent trans–cis and cis–trans photoisomerization processes are shown in Figure 4. The transmissivity was obtained as a ratio of intensities of the probe radiation (345 nm) detected by two photodiodes, one placed above and the other below the film. This ratio corresponds to the inverse transmittance of the films and subphase at 345 nm. Analogous to the solution, the inverse transmittance (and therefore also the absorption) decreases during irradiation with UV light and increases during irradiation with blue light. The effect of irradiation on the surface pressure and surface potential of the films is described in the Supporting Information.

The characteristic times for the transitions shown in Figure 4, obtained by fitting exponential functions to the experimental data, are 240 s for the trans–cis transition of GAzo, 190 s for the trans–cis transition of GAzo_3_, 430 s for the cis–trans transition of GAzo, and 40 s for the cis–trans transition of GAzo_3_.

Quite surprisingly, BAM imaging performed during the irradiation did not reveal any notable changes in the surface morphology of the film. Nevertheless, we detected evident changes in the surface pressure and surface potential of the film. Their analyses revealed that each of the modified properties changed at quite a different rate. The most rapid variations took place in optical transmittivity, while modifications of surface pressure were the slowest. The results can be found in the Supporting Information.

### 2.3 Photoisomerization in LB Films

**Figure 5:**
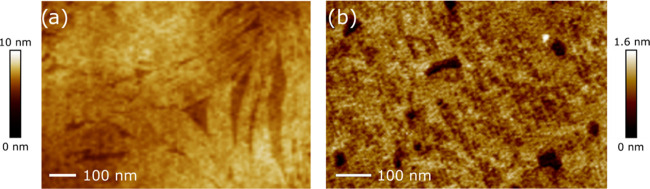
AFM images of surface morphology of GAzo (a) and GAzo_3_ (b) monolayers deposited onto silicon substrates.

Langmuir–Blodgett films were fabricated at deposition surface pressures of 20 mN/m for GAzo and of 33 mN/m GAzo_3_. Based on the atomic force microscopy (AFM) imaging, most homogeneous LB films were obtained at these values. AFM images of the films transferred onto silicon substrates are shown in Figure 5. The GAzo films exhibit a lamellar structure, while the GAzo_3_ films appear to be quite amorphous. The height of the lamellae in Figure 5a is approximately 1 nm, which is consistent with the expected monolayer thickness. The depth of the occasional holes found in the GAzo_3_ film is also approximately 1 nm. However, similar to the Langmuir films, we were unable to notice any evident changes in the surface morphology of the films when the films were irradiated with the UV or blue light. Similarly, there were also no significant differences observed between the films transferred with or without prior UV irradiation. This signifies that the isomerization reaction does not affect a close-packed surface arrangement of the molecules, which suggests that their packing features are determined mainly by the guanosine moiety.

To enable measurements of the UV–vis absorption spectra of the LB films, the films were deposited onto quartz substrates. Since it has been reported that the configuration state of the molecules acquired during the LB deposition can influence the fraction of molecules that are capable of isomerization after the deposition,^86^ we performed LB depositions of non-irradiated Langmuir films and also depositions of the films that were irradiated with UV light for 60 min prior to the deposition. In the following, the latter will be called cis-transferred LB films, while the former will be called trans-transferred LB films.

**Figure 6:**
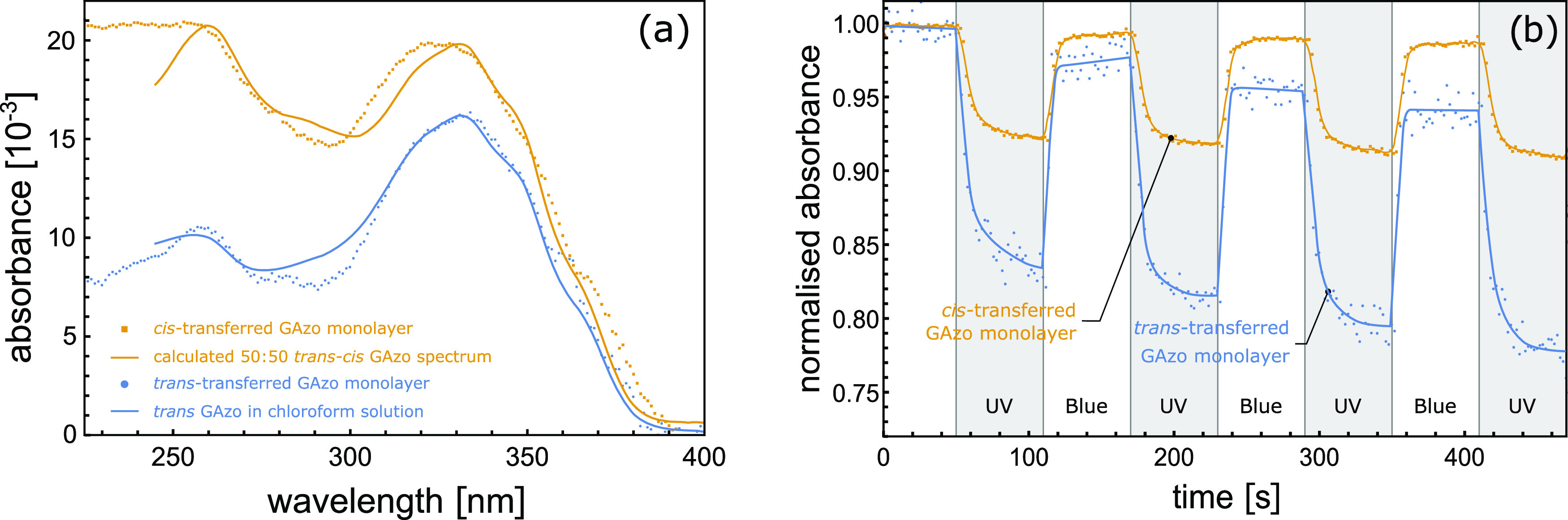
(a) Absorption spectra of cis-transferred (yellow) and trans-transferred (blue) LB films of GAzo and their comparison to the absorption spectrum of GAzo in solution. The cis-transferred monolayer was deposited after irradiating the film at the air–water interface with UV light for 60 min, while the trans-transferred film was deposited without any irradiation prior to the deposition. (b) Relative changes in absorbance (at λ = 330 nm) of LB films of GAzo on quartz substrates during subsequent irradiation with UV and blue light. Intervals of UV irradiation are shaded in gray. The type of irradiation is also marked at the bottom of the plot.

The UV–vis absorption spectra of LB films of GAzo and photoinduced changes in these spectra are shown in Figure 6. For the cis-transferred film, the principal absorption peak at around 330 nm is slightly blue-shifted with respect to the spectrum measured in solution (Figure 6a). A blue shift in the absorption spectrum is known to occur for azobenzene aggregates with dipole moments in the antiparallel arrangement. However, solvent interaction can also affect the exact position of the peak.^58^ The cis-transferred GAzo film has a significantly high absorbance at 250 nm as well, which is consistent with the cis configuration of azobenzene. In fact, the measured absorption spectrum corresponds to the shape of the spectrum that is obtained by summing up the solution absorption spectra of the *trans*- and *cis*-GAzo. This finding indicates that during the LB deposition of the cis-transferred film, about half of the molecules remain fixed in the cis configuration, possibly because cis–trans back-transition is sterically hindered by intramolecular and substrate–adsorbate interactions.

The difference in absolute absorbance values between the cis-transferred and the trans-transferred films indicates that either more molecules were deposited in the cis-transferred film or, since absorbance depends on the angle between light polarization and the molecular transition dipole moment,^58^ that orientation of azobenzene molecules in cis-transferred films is such that they absorb more light. Since the polarization of the light of the spectrophotometer lies in the plane of the substrate, this would mean that the angle between the transition dipole moments of the molecules and the plane of the substrate is, on average, smaller in the cis-transferred film than in the trans-transferred film.

Photoinduced changes in absorption of the LB films of GAzo at 330 nm are shown in Figure 6b. Both trans-transferred and cis-transferred films exhibit a lower irradiation-induced relative change in the absorbance than that measured in the solution: namely, 17% for the trans-transferred film and 7% for the cis-transferred film vs 60% obtained in solution (see Figure 8). The smaller change of absorbance in the cis-transferred film is consistent with the hypothesis that by the LB deposition about half of the molecules remain hindered in the cis state and consequently cannot contribute to the photoinduced modification of the absorption spectrum.

The characteristic times for the transitions shown in Figure 6, obtained by fitting exponential functions to the experimental data, are 8 s for the trans–cis transition of the trans-transferred GAzo film, 6 s for the trans–cis transition of the cis-transferred GAzo film, 2 s for the cis–trans transition of the trans-transferred GAzo film, and 4 s for the cis–trans transition of the cis-transferred GAzo film.

**Figure 7:**
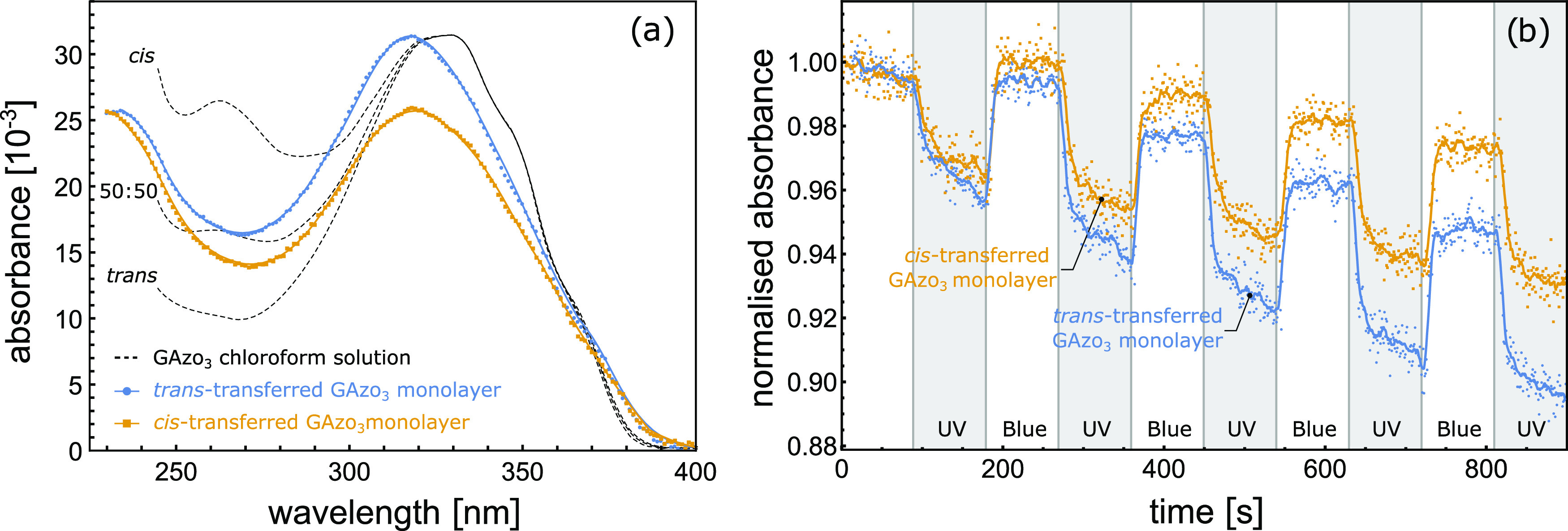
(a) Absorption spectra of cis-transferred (yellow) and trans-transferred (blue) LB films of GAzo_3_ and their comparison to the absorption spectrum of GAzo_3_ in solution. The absorption spectrum marked with “50:50” represents the shape obtained by summing both the trans and the cis solution spectra. All spectra in solution are normalized so that the height of the absorption peak at 330 nm is equal to the height of the corresponding peak in the trans-transferred monolayer. The cis-transferred monolayer was deposited after irradiating the film at the air–water interface with UV light for 60 min, while the trans-transferred film was deposited without prior irradiation. (b) Relative changes in absorbance (at λ = 330 nm) of LB films of GAzo_3_ on quartz substrates during subsequent irradiation with UV and blue light. Intervals of UV irradiation are shaded in gray. The type of irradiation is also marked at the bottom of the plot.

The UV–vis absorption spectra of LB-transferred GAzo_3_ films and photoinduced changes in these spectra are shown in Figure 7. In this case, the principal absorbance peaks of both trans-transferred and cis-transferred films are blue-shifted (Figure 7a). The ratio of absorbances between the two absorption peaks for the cis-transferred film is slightly lower than that for the trans-transferred film; however, the difference is not so pronounced as in the case of GAzo films. For GAzo_3_ films, the absorption spectrum of the monolayers does not correspond to any combination of the trans and cis spectra measured in chloroform solution: the ratio of absorbances between the peak at 230 nm and the valley at 270 nm is higher than that in the solution spectrum of *trans*-GAzo_3_, but, on the other hand, the spectra of the monolayers at the same time also do not exhibit the additional peak at 260 nm as observed in the solution spectrum of the *cis*-GAzo_3_. It is possible that when the molecules are compressed into a close-packed film the three azobenzene moieties begin to interact with each other, which affects the shape of their absorption spectrum. This conclusion is based also on the fact that no such blue shift was observed for the GAzo derivative that contains only one rather than three azobenzene groups, and hence, in the compressed GAzo films, azobenzene groups are less closely packed than those in the compressed GAzo_3_ films.

The amplitudes of the photoinduced changes in absorbance for both films (Figure 7b) are quite similar and are again much lower than that in solution (∼4 vs ∼40%), but much higher than it was measured in the Langmuir film at the air–water interface (∼4 vs ∼0.4%). For both films, the measured absorbance over time decreases, indicating some irreversible changes occurring in the material.

The characteristic times of the absorbance modifications shown in Figure 7b, obtained by fitting exponential functions to the experimental data, are 6 s for the trans–cis transition of the trans-transferred GAzo_3_ film, 12 s for the trans–cis transition of the cis-transferred GAzo_3_ film, 6 s for the cis–trans transitions of the trans-transferred GAzo_3_ film, and 7 s for the cis–trans transition of the cis-transferred GAzo_3_ film.

## 3 Discussion and Conclusions

Despite not having a chemical structure typical of the amphiphilic molecules, GAzo and GAzo_3_ form relatively stable Langmuir films at the air–water interface. The shapes of their isotherms are reminiscent of those observed by Haycraft et al. for so-called “bulge amphiphiles”, i.e., molecules with a large nonpolar group attached to the hydrophobic end of the hydrocarbon chain.^87,88^ They also observed a plateauing of the surface pressure after the collapse, accompanied by the appearance of bright spots in the BAM images of the film, which in time coalesced into larger formation. Moreover, they also observed cases of large hysteresis in the compression–expansion cycles.

Observations by BAM revealed unique structures of Langmuir films of the two investigated molecules. GAzo molecules immediately formed multilayered structures that became denser when the surface area of the film was reduced. In contrast, GAzo__3__ molecules formed a flake-like solid phase, which did not disintegrate after the film was expanded. It is tempting to attribute the rigidity of this phase to hydrogen bonding between the guanosine molecules. However, similar dense structures were also observed in films of azo-functionalized polymers^89^ as well as in films of some proteins.^90,91^ So, the nature of the intriguing solidification of the GAzo__3__ films still remains an open question.

The observed irradiation-induced changes in the absorption spectra of the films demonstrate that the investigated molecules can undergo a reversible photoisomerization both at the air–water interface and also after their LB deposition onto solid substrates. Therefore, they provide an attractive possibility that isomerization of the azobenzene group can be used to regulate their base pairing in various thin-film configurations.^81^ This effect will be investigated in a separate study. However, the fraction of the molecules in the films that are switched upon irradiation is considerably smaller than the fraction of the molecules switched in solution. Several papers have reported observations of photoinduced changes in the film structure of photosensitive molecules using AFM.^86,92,93^ However, in our case, regardless of evident modifications in the optical absorption/transmission properties of the films, no change in their surface morphology could be resolved.

The characteristic time τ for photoinduced isomerization is proportional to the intensity *I* of the light used for irradiation^94^
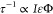
where ε is the molar absorption coefficient of the molecules and Φ is the quantum yield for the specific molecular transition. Because of large variations in *I* among different setups, it is difficult to make a meaningful comparison of the obtained values of τ for different types of films and for the solution. But, the ratio of τ obtained for different molecules measured with the same setup is independent of *I*, so this makes more sense. Table 1 shows the ratio τ_GAzo_/τ_GAzo_3__ measured in different experimental configurations. One can find out that there is no systematic rule dictating a relationship between the characteristic times for isomerization of GAzo_3_ molecules (τ_GAzo_3__) and the characteristic times for isomerization of GAzo molecules (τ_GAzo_). In solution, τ_GAzo_3__ is larger than τ_GAzo_ for both transitions (trans–cis and cis–trans), while in Langmuir films the situation is just the opposite. In trans-transferred LB films, for cis–trans transition, τ_GAzo_3__ is larger than τ_GAzo_, while the reverse is valid for the trans–cis transition. In cis-transferred LB films, the values of τ_GAzo_3__ for both transitions are again larger than the values of τ_GAzo_. This suggests that in the investigated systems both the orientation of molecular transition dipole moments and the quantum yield of the transitions are quite different.

**Table 1:** Ratio of the Characteristic Times of Photoinduced Cis–Trans and Trans–Cis Transitions for GAzo and GAzo_3_ Molecules in Various Investigated Systems

	τ_GAzo_/τ_GAzo_3__	
	trans–cis	cis–trans
solution	2.2	3.0
Langmuir film	0.8	0.1
trans-transferred LB film	0.8	3.0
cis-transferred LB film	2.0	1.8

In summary, we synthesized two novel azo-functionalized guanosine derivatives and demonstrated that despite the fact that they do not possess a molecular structure typical of amphiphilic molecules, they do form stable Langmuir films at the air–water interface. The two compounds can undergo repeated, reversible photoisomerization both in films at the water surface and in films transferred to solid substrates. However, when compared to the isomerization efficiency in solution, the isomerization efficiency in the films is significantly reduced. The UV–vis spectra of the GAzo film transferred after irradiation with UV light also revealed that part of the molecules remain locked in the cis configuration and were unable to undergo cis–trans isomerization.

Our results demonstrate that DNA nucleobases functionalized with relatively simple azobenzene moieties can form stable monomolecular films that can undergo repetitive photoisomerization. They are, hence, suitable candidates for the fabrication of photoactive 2D materials that provide all beneficial functionalities of DNA-based compounds, such as selective recognition, biocompatibility, high surface charge, etc.

## 4 Materials and Methods

### 4.1 Azo-Functionalized Guanosine Derivatives

**Figure 8:**
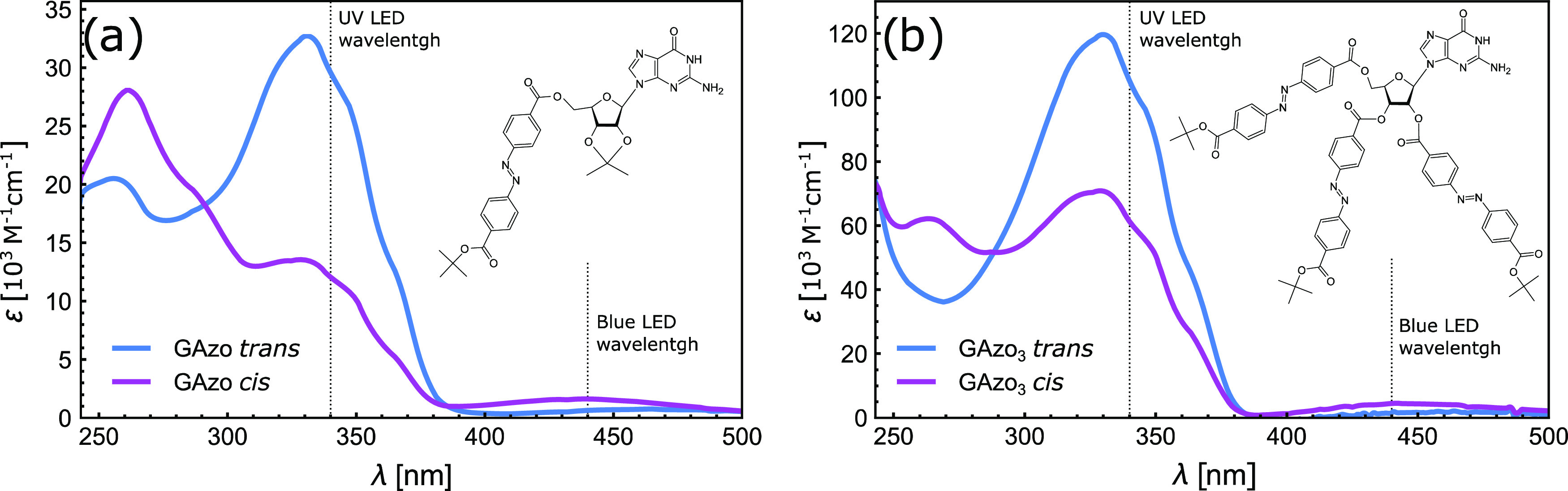
UV–vis absorption spectra of GAzo (a) and GAzo_3_ (b) in chloroform solution. The chemical structures of the molecules are shown in the insets. Vertical dotted lines indicate the wavelengths of the light-emitting diode (LED) light sources used to induce photoisomerization. The trans spectra of the molecules were recorded after the solutions had been kept in the dark for several days, and the cis spectra were recorded after the solutions were irradiated with UV light until a photostationary state was reached.

The molecular structures of the two azo-functionalized guanosine derivatives used in this work are shown in the insets of Figure 8a,b: we will refer to these molecules as GAzo and GAzo__3__, respectively. GAzo has a single azobenzene moiety attached at the 5′ position on the ribose (Figure 8a), while GAzo__3__ has three azobenzene moieties attached at positions 2′, 3′, and 5′ on the ribose (Figure 8b). Details on the synthetic procedures and compound characterization are reported in the Supporting Information.

The absorption spectra of the molecules in chloroform solution are shown in Figure 8. They demonstrate that trans–cis isomerization can be generated by irradiation with UV light (λ = 340 nm), while cis–trans isomerization can be induced by light in the visible range of the spectrum (λ = 440 nm).

### 4.2 Langmuir Film Preparation

Langmuir films investigated in this work were prepared in a KSV Nima KN3002 Langmuir–Blodgett trough with a maximum available surface area of 549 cm^2^ (7.5 cm × 73.2 cm) and two symmetrically driven hydrophobic barriers for monolayer compression. All measurements were performed at room temperature (23 ± 1 °C). The trough was filled with ultrapure water (Adrona Onsite+). Before the deposition of guanosine molecules, the purity of the water subphase was assessed by measuring the change in surface pressure during the standard compression/expansion cycle and monitoring the surface structure by Brewster angle microscopy (BAM). A total change in surface pressure of less than 0.5 mN/m was considered satisfactory.

The surface pressure was measured by the Wilhelmy plate method using paper plates purchased from Biolin Scientific. The surface potential was measured using a KSV NIMA SPOT device. The trough was also equipped with a leveling tool to counteract the loss of water from the trough due to evaporation.

The spreading solution of guanosine derivatives was added to the air–water interface one drop at a time using a glass syringe (Hamilton). After spreading, the film was allowed to relax for 30 min in a noncompressed state to ensure that all of the solvent had evaporated and internal equilibrium had been established. Then, the barriers were compressed at a constant rate of 5 mm/min (3.75 cm^2^/min). When the target surface area or pressure was reached, the film was left to relax for another 30 min to reach a new equilibrium before optical irradiation or deposition to a solid substrate was initiated.

The LB films were deposited either onto quartz substrates for measurements of the UV–vis absorption spectra or onto silicon substrates for surface imaging with atomic force microscopy (AFM). Quartz substrates polished to the roughness of less than 1 nm and cut to pieces with a size of 25 mm × 10 mm were purchased from Solis Beijing Tech, while test-grade silicon wafers were purchased from University Wafer and manually cut to pieces of 25 mm × 10 mm in size. The retraction speed of the dipper in all LB depositions was 0.5 mm/min.

### 4.3 UV–Vis Absorption Measurements

The UV–vis absorption spectra of solutions placed in quartz cuvettes and of LB films deposited onto quartz substrates were measured using a commercial UV–vis spectrophotometer (Hewlett Packard 8453). Measurements of changes in light absorption of the Langmuir films at the air–water interface were performed by a custom-made setup built around the LB trough with a sapphire window installed at its bottom. A UV LED (Thorlabs M340L4, peak wavelength at 345 nm, full width at half-maximum (FWHM) = 10 nm) was positioned above the LB trough and acted as the light source for the measurements. The emitted light passed through the Langmuir film, the subphase, and the sapphire window. Then, it was detected by a photodiode. A second photodiode was positioned above the LB trough to record the reference intensity of the light source.

### 4.4 Optical Irradiation System

The trans–cis transition was induced by irradiating the samples with light from one or multiple UV LEDs (Zhuhai Tianhui Electronic Co., TH-UV340T3WA, peak wavelength at 340 nm, FWHM = 10 nm, 65 mW average total radiative power), while the reverse cis–trans transition was induced using blue LEDs (Chanzon, peak wavelength at 440 nm, FWHM = 20 nm, 500 mW average total radiative power). These two wavelengths correspond to the absorption peaks of trans and cis isomers of GAzo and GAzo_3_ molecules (see Figure 8).

To be able to irradiate solutions and LB films during measurements of their absorption spectra, we constructed a special holder for cuvettes and quartz substrates. It allowed us to irradiate the sample with actinic light from the sides, while the light beam of the spectrophotometer entered the samples from the front.

For irradiation of the films at the air–water interface, two arrays of four UV LEDs were positioned above the water surface, while four blue LEDs illuminated the water surface from the side. During AFM imaging, the samples were irradiated by LEDs positioned next to the scanning head.

### 4.5 Brewster Angle Microscopy and Atomic Force Microscopy

Brewster angle microscopy (BAM) of the surface of the films was performed with the built-in device of an Accurion EP3se ellipsometer, using the internal laser light source with a wavelength of 658 nm. AFM images of the films were obtained with a Veeco Dimension 3100 AFM using tapping mode.

## Supporting Information Available

Description of the synthesis of GAzo and GAzo_3_; and comparison of the behavior of surface pressure, surface potential, and light absorption of Langmuir films during photoisomerization (PDF)
