# Supplementary material for: Reversible Photoisomerization in Thin Surface Films from Azo-Functionalized Guanosine Derivatives
Source: ACS Omega. 2021 Jun 7;6(23):15421–30. doi: 10.1021/acsomega.1c01879 (PMC8210406; doi:10.1021/acsomega.1c01879)
Supplement: Supplementary file 1 — ao1c01879_si_001.pdf [file ao1c01879_si_001.pdf]

SUPPORTING INFORMATION

"REVERSIBLE PHOTOISOMERIZATION IN THIN SURFACE FILMS FROM AZO-FUNCTIONALIZED GUANOSINE DERIVATIVES"

*Matjaž Ličen[1], Stefano Masiero[2], Silvia Pieraccini[2], Irena Drevenšek Olenik[1,3]**

[1] University of Ljubljana, Faculty of Mathematics and Physics, Jadranska 19, 1000 Ljubljana, Slovenia

[2] Alma Mater Studiorum – Università di Bologna, Dipartimento di Chimica "Giacomo Ciamician", Via San Giacomo 11, I-40126 Bologna, Italy

[3] Department of Complex Matter, Jožef Stefan Institute, Jamova 39, SI 1000 Ljubljana, Slovenia

*irena.drevensek@ijs.si

TABLE OF CONTENTS:

Synthesis of GAzo and $GAzo_3$ S2

Comparison of surface pressure, surface potential, and light absorption of Langmuir films during photoisomerization S9

SYNTHESIS OF GAZO AND GAZO$_3$

General methods. All reactions requiring anhydrous conditions were carried out under dry argon atmosphere in oven-dried glassware. Macherey-Nagel Polygram silica gel plates (layer thickness 0.20 mm) were used for TLC analyses. Column chromatography was performed on Geduran silica gel 60 (40-63 μm). Reagents and solvents, including dry solvents, were purchased from Sigma-Aldrich or TCI. Electrospray ionization mass spectra were obtained from methanol solutions in either positive or negative mode with Micromass ZMD 4000 or ZQ-4000 instruments. HRMS spectra were recorded on a Waters Xevo G2-XS QTof system. Nuclear magnetic resonance spectra were recorded on Varian Inova (600, 400 or 300 MHz) spectrometers and referenced to the residual solvent resonance (

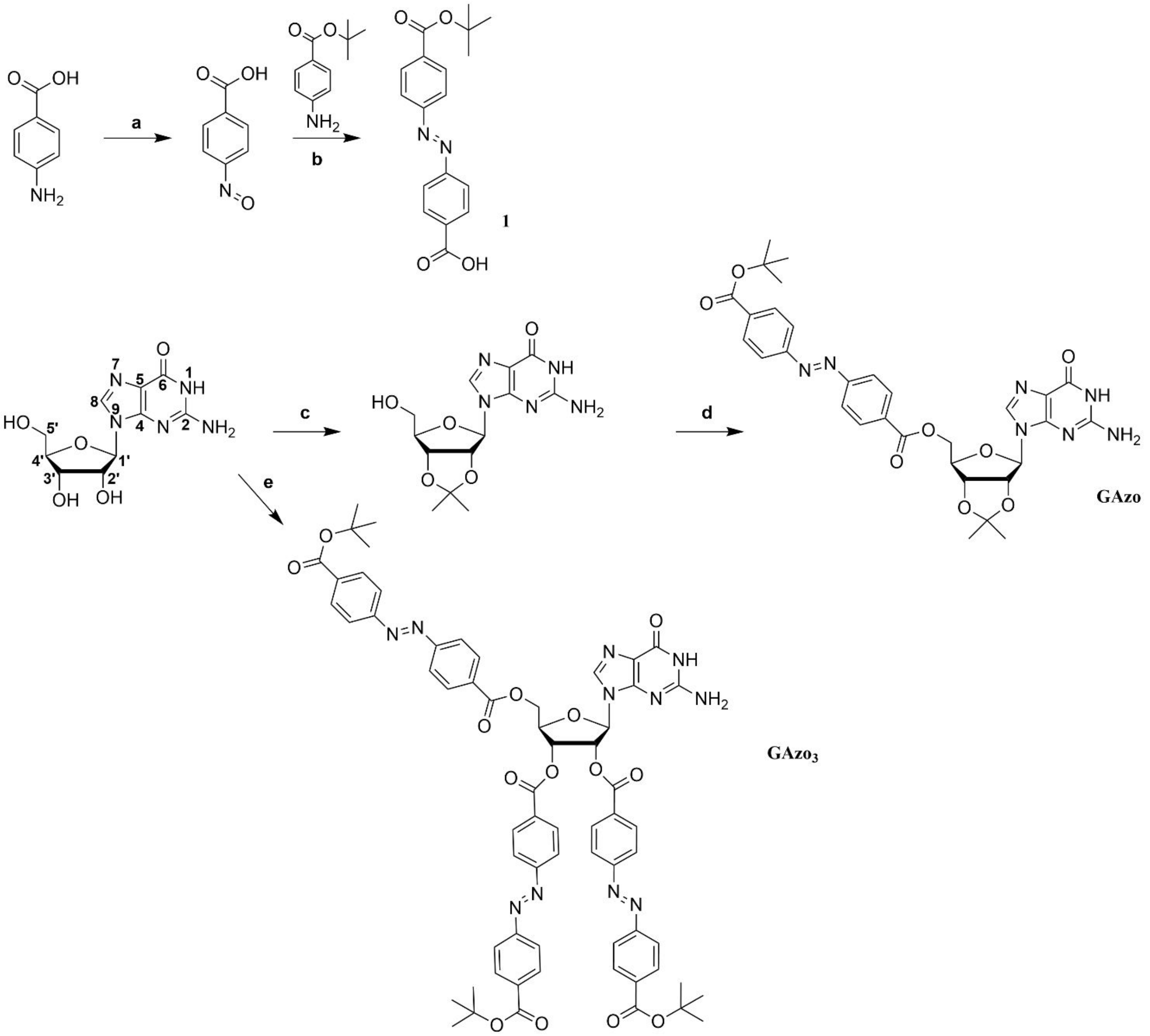

Scheme S1: synthesis of **GAzo** and **GAzo$_3$**. **a**) Oxone, DCM/$H_2$0, rt; **b**) DMSO/AcOH, rt; **c**) *i*-acetone, $HClO_4$, rt; *ii*-NH_3/H_2O, rt; **d**) **1**, DCC, DMF, rt; **e**) *i*-**1**, $MeSO_2Cl$, Et_3N, THF, 0°C; *ii*-DMAP, rt.

4-{(E)-[4-(tert-butoxycarbonyl)phenyl]diazenyl} benzoic acid **1**

4-Nitrosobenzoic acid[1] (1.057 g, 7.0 mmol) was suspended in a 1:1 mixture of DMSO and acetic acid (40+40 mL). t-Butil-4-aminobenzoate (1.62 g, 8.4 mmol) was then added and the mixture was immersed in an ultrasonic bath for 10 min. The finely dispersed suspension was stirred at rt and the reaction was monitored by TLC (CH_2Cl_2/MeOH 95:5). After 24h the orange suspension was filtered and the solid was purified by column chromatography on silica (CH_2Cl_2/MeOH 95:5). The isolated product was further purified by crystallization from EtOH, affording the acid **1** (0.80 g, 35% yield) as a pink-orange solid.

Rf_{trans} = 0.43 (CH_2Cl_2/MeOH 95:5).

ESI-MS (*m/z*): 324.9 $[M-H]^-$.

HR-MS: calcd. for $C_{18}H_{18}N_2O_4$, *m/z* 326.1267; found, *m/z* 326.1268.

^{1}H-NMR δ(dmso-d_6): 1.58 (s, 9H, tBu), 7.99-8.02 (m, 4H, ArH), 8.11-8.17 (m, 4H, ArH), 13.33 (bs, 1H, COOH) ppm.

^{13}C-NMR δ(dmso-d_6): 28.23 (CH_3), 81.87 (C), 123.20 (CH), 123.28 (CH), 130.89 (CH), 131.07 (CH) 134.18 (C), 154.36 (C), 154.64 (C), 164.71 (C) ppm.

5'-O-[(E)- 4-(tert-butoxycarbonyl)phenyl)diazenyl)benzoyl]-2',3'-O-isopropilidene guanosine (**GAzo**)

To a stirred solution of acid **1** (0.503 g, 1.5 mmol) in DMF (10 mL) were added 0.639 g, (3.1 mmol) of N,N′-dicyclohexylcarbodiimide. After 30 min, 2',3'-O-isopropylidene guanosine[2] (0.417 g, 1.3 mmol) and 4-(dimethylamino)pyridine (0.16 g, 1.3 mmol) were added. Progress of the reaction was monitored by TLC (CH_2Cl_2/MeOH 9:1). The reaction mixture was stirred for 42 h at r.t. then the solvent was distilled off under reduced pressure. The crude reaction mixture was partitioned between CH_2Cl_2 and sat. Na_2CO_3. The aqueous phase was washed several times with CH_2Cl_2 and the combined organic fractions were dried over $MgSO_4$. Solvent was removed by distillation and the residue was purified by column chromatography (CH_2Cl_2/MeOH, gradient from 99:1 to 9:1). The product thus obtained was further crystalized from MeOH, affording 0.349 g (43%) of the title compound as an orange solid.

Rf_{trans} = 0.55 (CH_2Cl_2/MeOH 9:1).

ESI-MS (*m/z*): 630.0 $[M-H]^-$; 632.1 $[M+H]^+$; 654.1 $[M+Na]^+$.

HR-MS: calcd. for $C_{31}H_{33}N_7O_8$, *m/z* 631.2391; found, *m/z* 631.2393.

^{1}H-NMR δ(dmso-d_6): 1.35 (s, 3H, CH3), 1.55 (s, 3H, CH3), 1.58 (s, 9H, tBu), 4.42-4.47 (m, 2H, H5', H4'), 4.56-4.61 (m, 1H, H5'), 5.30-5.33 (m, 2H, H3', H2'), 6.08 (s,1H, H1'), 6.56 (bs, 1H, NH_2), 7.86 (s, 1H, H8), 8.01-8.04 (m, 4H, ArH), 8.12-8.15 (m, 4H, ArH), 10.71 (bs, 1H, NH) ppm.

^{13}C-NMR δ(dmso-d_6): 25.86 (CH3), 27.52 (CH3), 28.22 (CH3, tBu), 65.53 (CH_2, 5'), 81.49 (CH, 3'), 81.91(C, tBu), 84.24 (CH, 2'), 84.71 (CH, 4'), 88.85 (CH, 1'), 113.87 (C, CMe_2), 117.50 (C, C5), 123.37 (CH, ArH), 123.41 (CH, ArH), 130.89 (CH, ArH), 131.20 (CH, ArH), 132.27 (C), 134.35 (C), 136.69 (CH, C8), 150.92 (C, C4), 154.16 (C), 154.58 (C, ArH), 154.85 (C, ArH), 157.15 (C), 164.68 (C, COOtBu), 165.22 (C, COO) ppm.

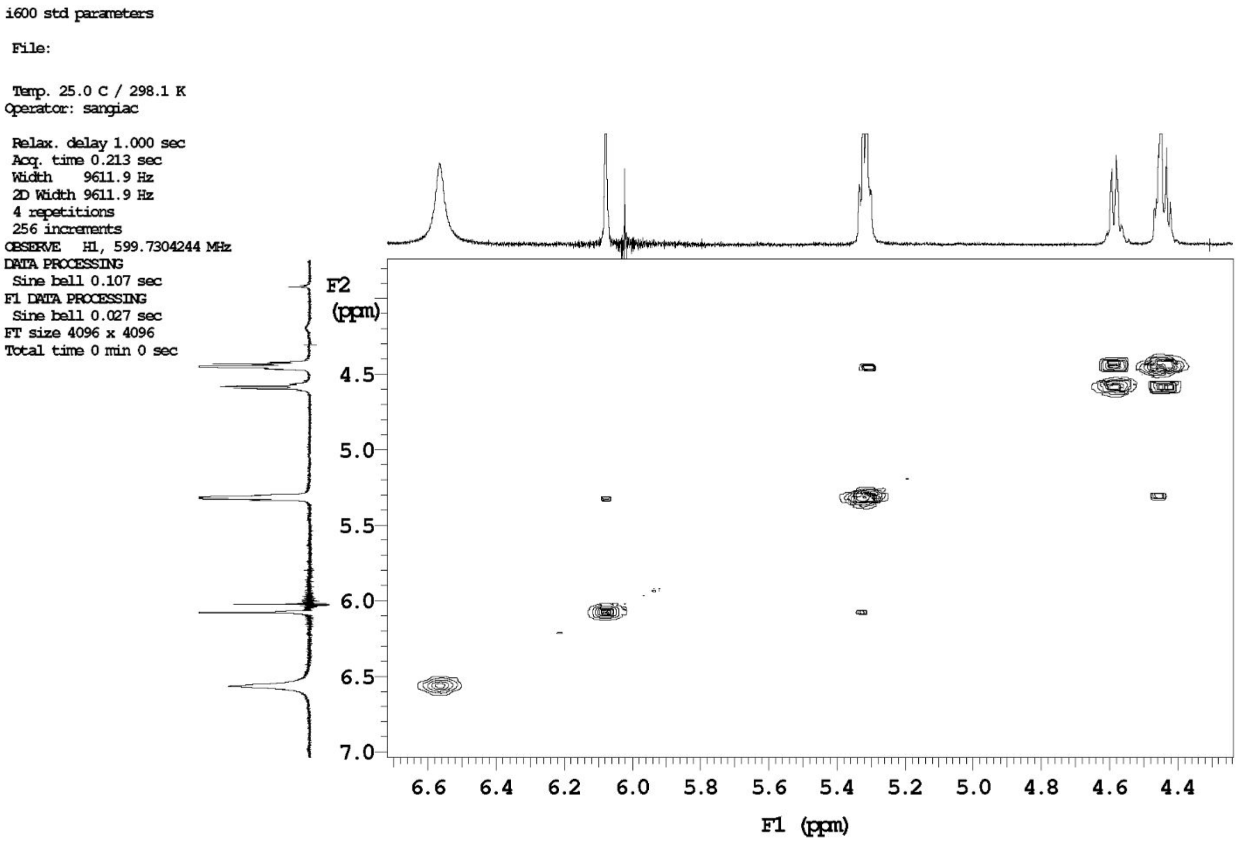

Figure S1: Sugar region of the gCOSY spectrum of **GAzo** in dmso-d_6.

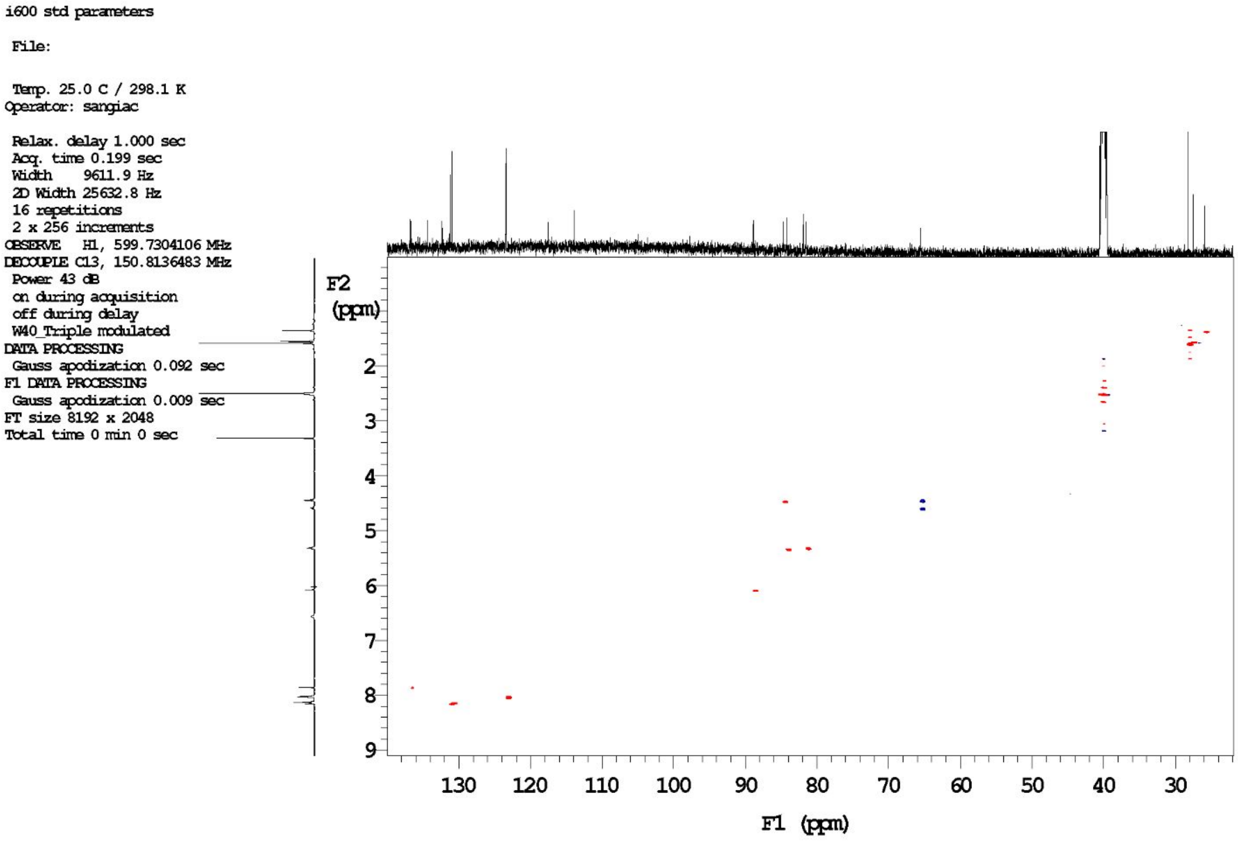

Figure S2: gHSQC spectrum of **GAzo** in dmso-d_6.

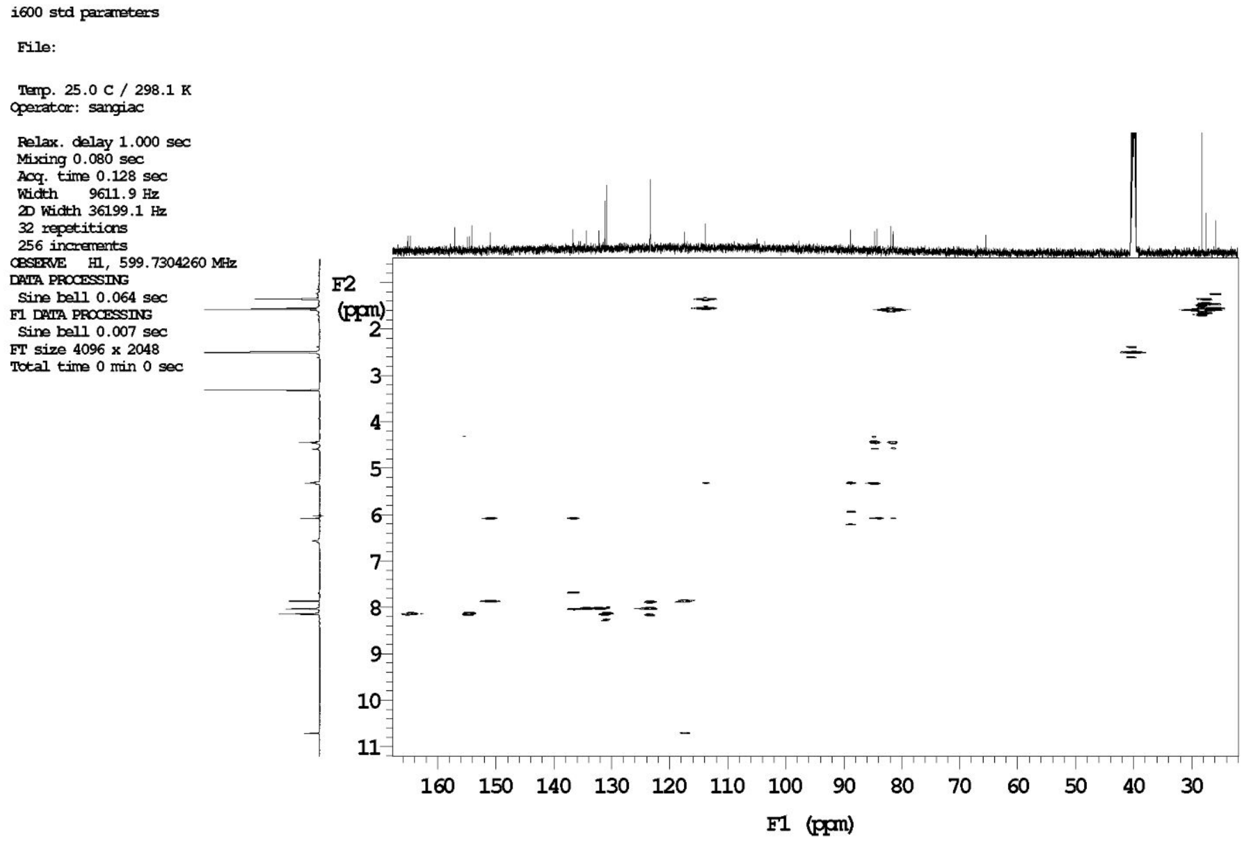

Figure S3: gHMBC spectrum of **GAzo** in dmso-d_6.

<u>2',3',5' –tri-O-[(E)- 4-(tert-butoxycarbonyl)phenyl)diazenyl)benzoyl]guanosine</u> (**GAzo$_3$**)

Acid **1** (0.351 g, 1.08 mmol) was dissolved in THF (10 mL) and the resulting solution was cooled to 0°C. Triethylamine (280 µL, 2.0 mmol) and methanesulfonyl chloride (88 µL, 1.1 mmol) were added and the mixture was stirred for 1 h at 0°C then allowed to warm to r.t. Guanosine (85 mg, 0.30 mmol) and a catalytic amount of 4-(dimethylamino)pyridine were added and the reaction was monitored by TLC (CH_2Cl_2/MeOH 96:4). After 48 h the solvent was removed by distillation under reduced pressure. The crude reaction mixture was partitioned between $CHCl_3$ and sat. Na_2CO_3. The aqueous phase was washed several times with $CHCl_3$ and the combined organic fractions were dried over $MgSO_4$. Solvent was removed by distillation and the residue was purified by column chromatography ($CHCl_3$/MeOH, gradient from 99:1 to 97:3). The product thus obtained was further crystalized from MeOH, affording 0.176 g (48%) of the title compound as a bright orange solid.

Rf_{trans} = 0.33 ($CHCl_3$/MeOH 97:3).

ESI-MS (*m/z*): 1206.6 $[M-H]^-$; 1208.6 $[M+H]^+$; 1230.5 $[M+Na]^+$.

HR-MS: calcd. for $C_{64}H_{61}N_{11}O_{14}$, *m/z* 1207.4399; found, *m/z* 1207.4395.

^{1}H-NMR δ(CD_2Cl_2/dmso-d_6): 1.582 (s, 9H, tBu), 1.585 (s, 9H, tBu), 1.590 (s, 9H, tBu), 4.72-4.75 (m, 1H, H5'), 4.85-4.87 (m, 1H, H4'), 4.91-4.94 (m, 1H, H5'), 5.71 (bs, 2H, NH_2), 6.26 (d, J=4.0, 1H, H1'), 6.40 (dd, J=5.8, 4.0, 1H, H2'), 6.43 (t, J=5.8, 1H, H3'), 7.66 (s, 1H, H8), 7.89-7.95 (m, 12H, ArH), 8.08-8.12 (m, 8H, ArH), 8.13-8.15 (m, 2H, ArH), 8.17-8.19 (m, 2H, ArH), 10.76 (bs, 1H, NH) ppm.

^{13}C-NMR δ (CD_2Cl_2/dmso-d_6): 27.82 (CH3, tBu), 63.66 (CH_2, 5'), 71.41 (CH, 3'), 73.89 (CH, 2'), 79.45 (CH, 4'), 81.49 (C, tBu), 86.98 (CH, 1'), 118.36 (C, C5), 122.68 (CH, ArH), 122.91 (CH, ArH), 130.32 (CH, ArH), 130.72 (CH, ArH), 130.78 (CH, ArH), 130.88 (CH, ArH), 131.65 (C), 134.56 (C), 134.66 (C), 136.22 (CH, C8), 150.70 (C, C4), 153.75 (C), 154.50 (C), 154.56 (C), 155.13 (C), 155.29 (C), 155.36 (C), 164.63 (COO), 164.72 (COO), 165.47 (COO) ppm.

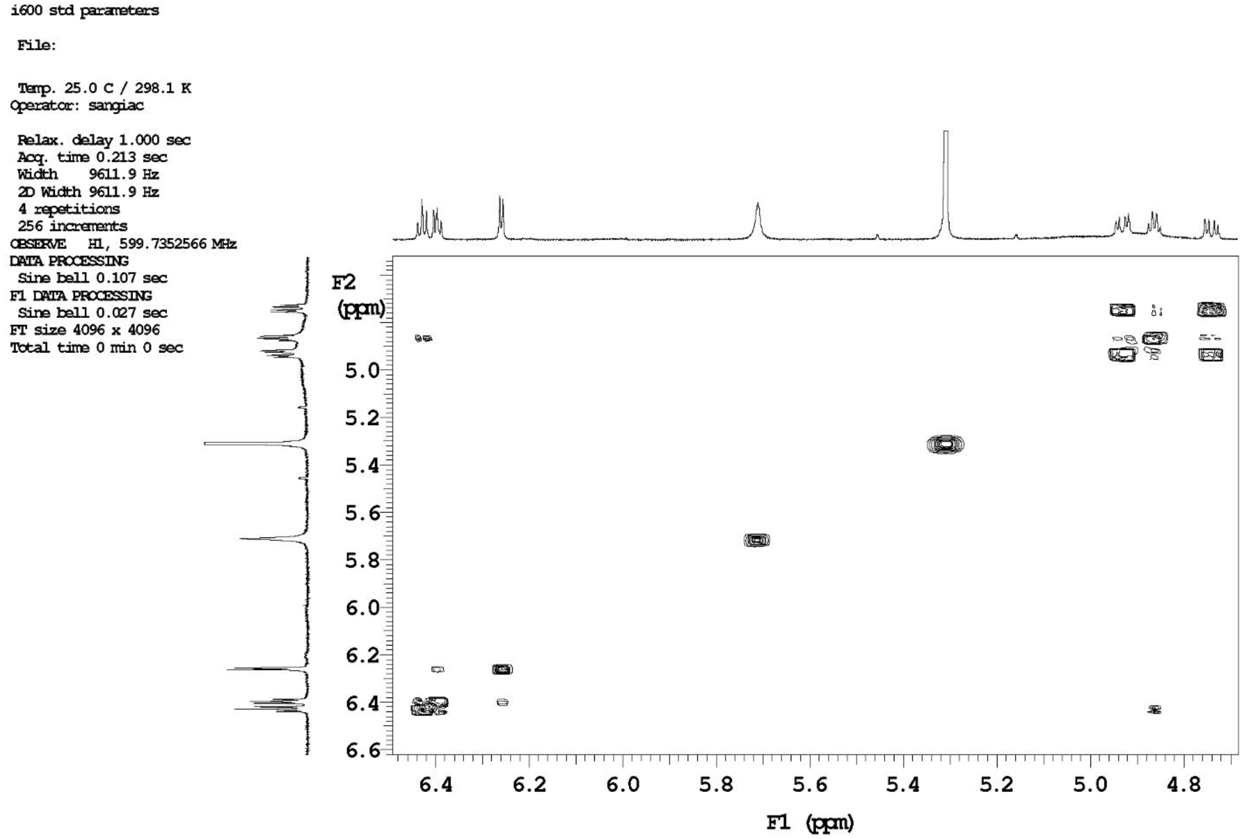

Figure S4: Sugar region of the gCOSY spectrum of **GAzo$_3$** in CD_2Cl_2/dmso-d_6.

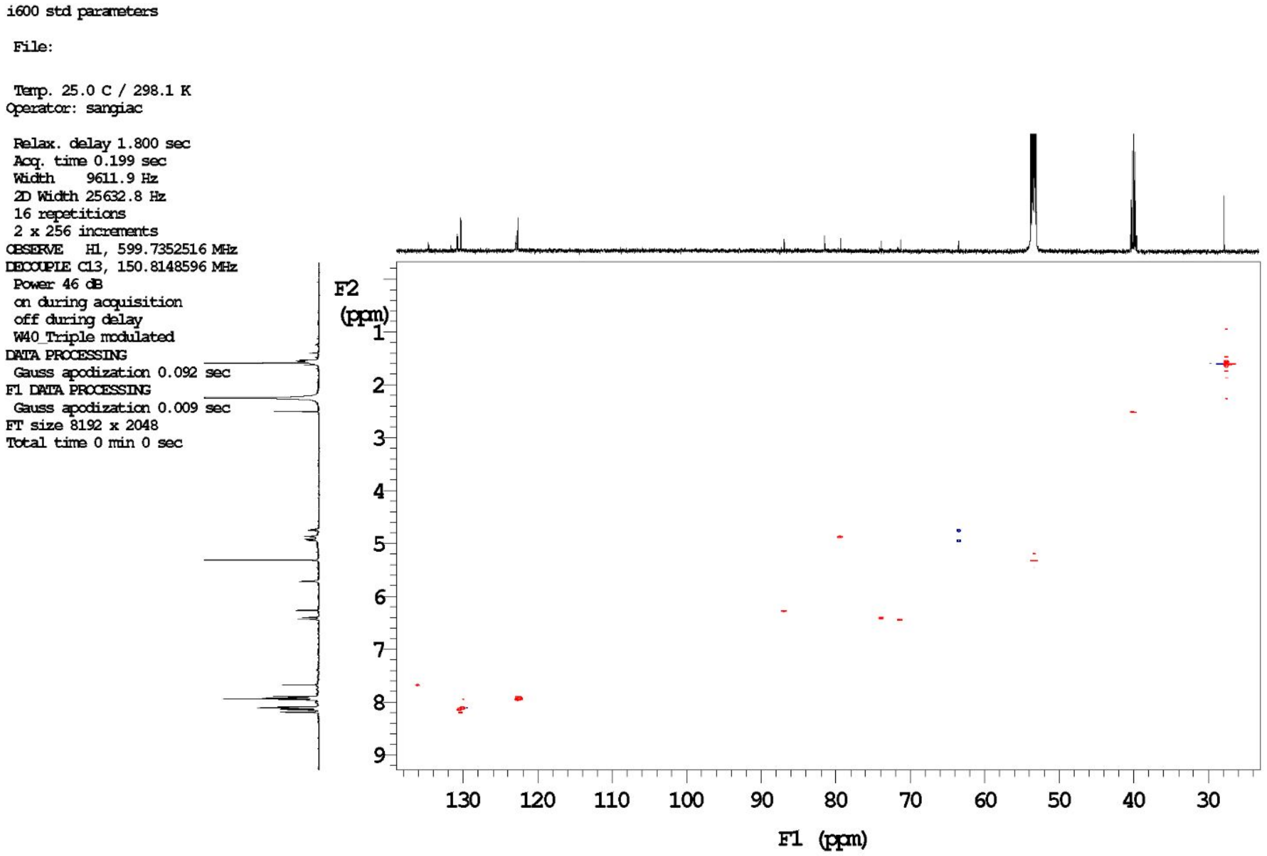

Figure S5: gHSQC spectrum of **GAzo$_3$** in CD_2Cl_2/dmso-d$_6$.

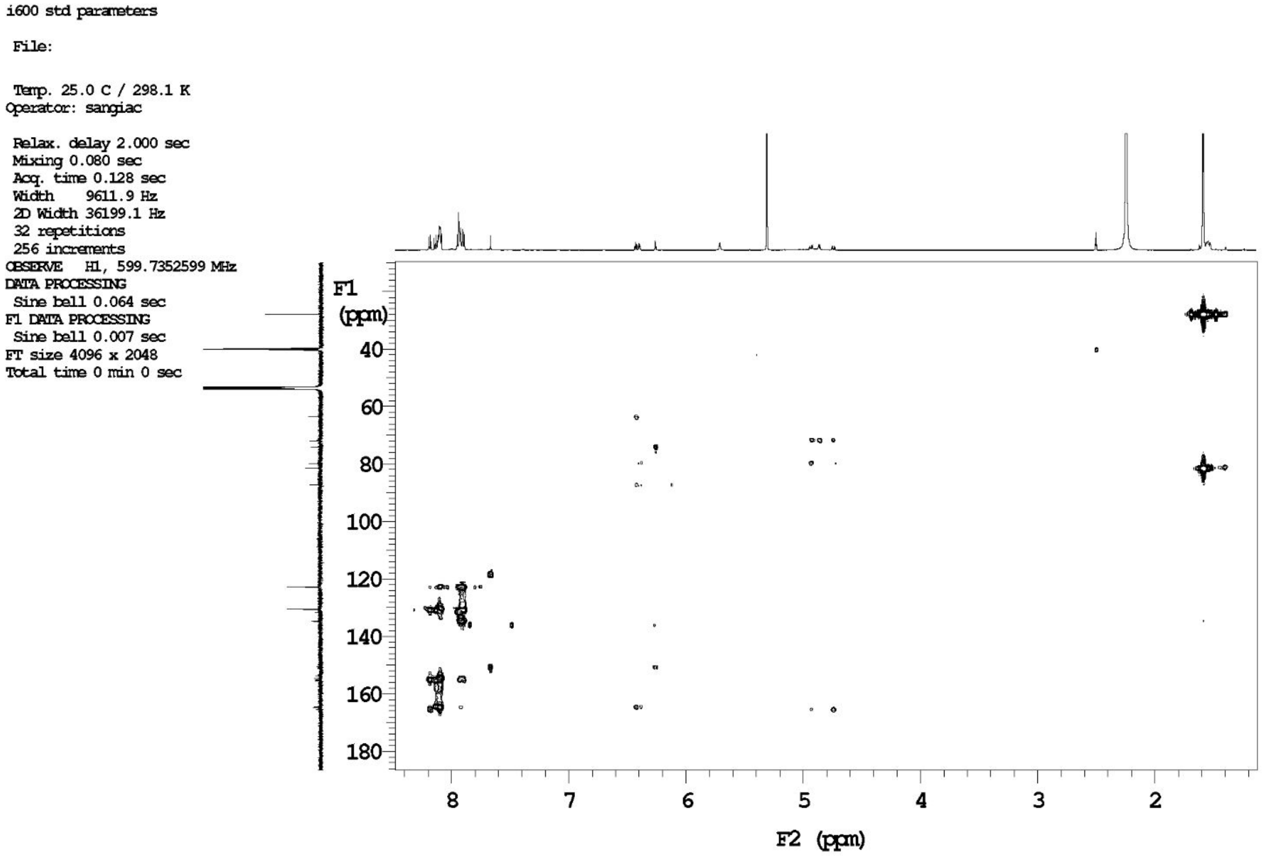

Figure S6: gHMBC spectrum of **GAzo$_3$** in CD_2Cl_2/dmso-d$_6$.

COMPARISON OF SURFACE PRESSURE, SURFACE POTENTIAL, AND LIGHT ABSORPTION OF LANGMUIR FILMS DURING PHOTOISOMERIZATION

Langmuir films from GAzo and $GAzo_3$ were prepared by depositing 75 µL (GAzo) or 60 µL ($GAzo_3$) of 1 mM chloroform solution to the air water surface and compressing the barriers so that the final surface area was equal to 177.5 cm^2. This resulted in mean molecular areas of 39 $Å^2$ in the case of GAzo film and 49 $Å^2$ in the case of $GAzo_3$ film.

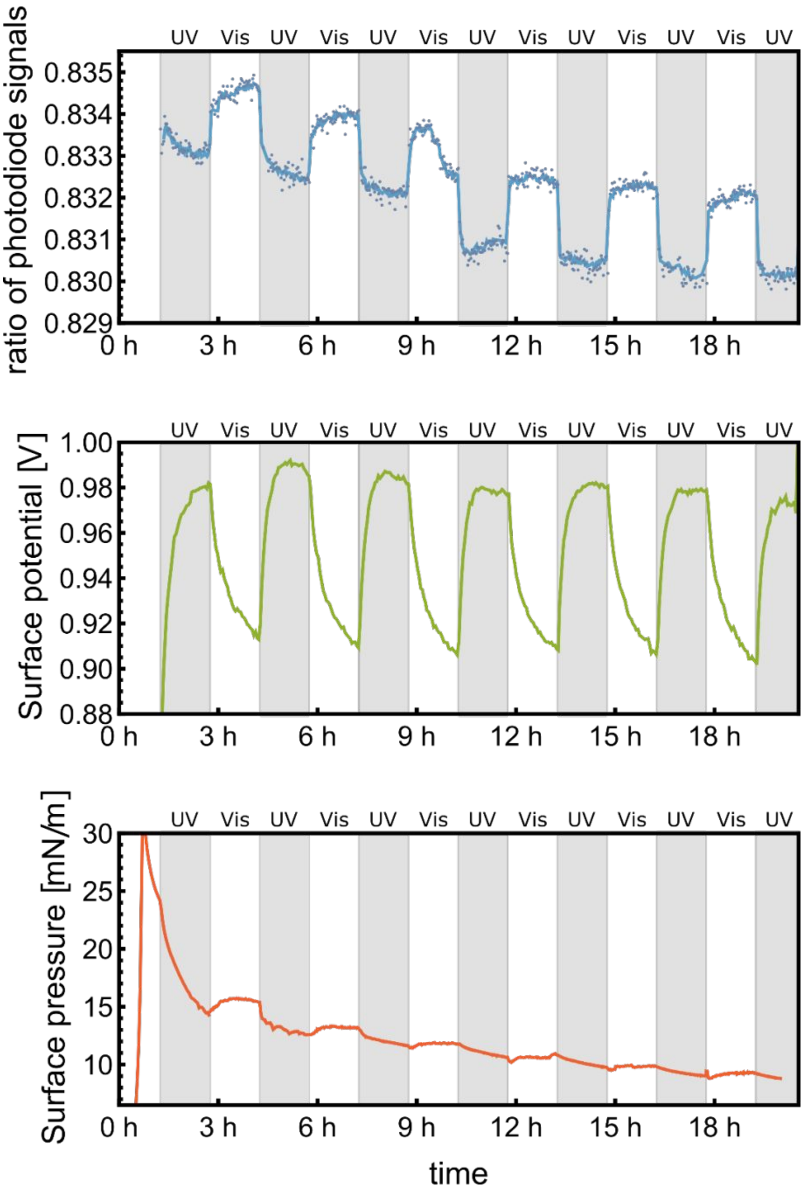

Figure S7: Changes in light absorption (blue), surface potential (green), and surface pressure (red) of a GAzo film during irradiation with UV and blue light. The intervals with gray background indicate UV irradiation, while white background indicates irradiation with visible light. Light absorption is shown in terms of the change of the ratio of the voltage signals recorded on the two photoiodes. A brigther blue line is drawn on top of the data points for the photoiode signal: this represents the same data but filtered to reduce measurement noise.

The recorded changes in light absorption, surface potential, and surface pressure in GAzo film during irradiation with blue and UV light are shown in Figure S. While irradiation induced a significant drop in surface pressure (from 30 mN/m at the end of compression to below 10 mN/m at the end of the measurement), surface potential and absorbance both remained mostly constant – the slight drop in the signal on the photodiode seen in Figure S amounts to only a 0.3 % increase in signal with respect to the start of the measurement. Had the drop in surface pressure been caused by the loss of molecules into the subphase, we would expect a proportional drop in absorption of light and surface potential. The fact that this was not observed leads us to believe that the drop in surface pressure is not a consequence of the dissolution of molecules in the subphase but rather the consequence of the molecules rearranging themselves at the film surface, possibly forming multilayered structures. This is also consistent with the continued evolution of the appearance of the water surface after compression, as observed under BAM.

A single cycle of blue and UV irradiation is shown in greater detail in Figure S. The data in this image are fitted with exponential functions to better illustrate the characteristic times for the change in each of the measured quantities. A linear drift term was added when fitting the data for surface pressure to account for the drop in surface pressure over the entire course of the measurement.

When the film is irradiated with blue light, the change in light absorption happens the fastest, with surface pressure and surface potential changing at slower rates. During UV irradiation, however, surface pressure follows the change in absorption, while the change in surface potential still lags behind the two. The fact that the fitted characteristic time for the change in surface pressure is actually shorter than the one for absorption is likely a consequence of the fact that a simple exponential function with a linear drift term does not accurately describe the behavior of surface pressure during irradiation[3].

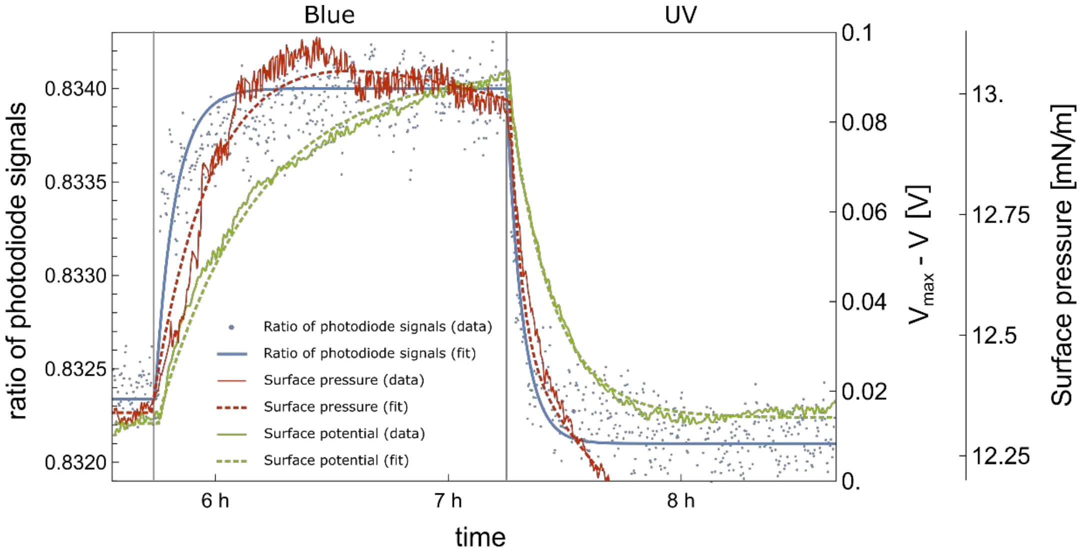

Figure S8: Surface pressure, surface potential, and light absorption of GAzo Langmuir film during a single cycle of blue and UV irradiation. The dashed lines are exponential fits – the obtained characteristic times are written next to the lines. In the case of surface pressure, an additional linear drift term was added to the fitting function: in this case, the dotted line represents the fit without the added linear drift term.

Photoinduced changes in light absorption, surface potential, and surface pressure in a $GAzo_3$ film are shown in Figure S9. Similar observation as for the data measured in GAzo films can be made here as well, however, there is a slight downward trend in surface potential, consistent with loss of molecules from the film

surface. However, the relative change in surface potential is only 4%, which is still small in comparison to the much larger drop in surface pressure.

Figure S10 shows a more detailed view of a single blue and UV irradiation cycle of $GAzo_3$ Langmuir film. Just as in the case of the GAzo film, the change in light absorption occurs at a faster rate than the change in surface pressure and surface potential. The characteristic time for the change in light absorption during irradiation with blue light is remarkably short, especially when compared to the characteristic time for the change in surface potential: 40 s vs. 1300 s.

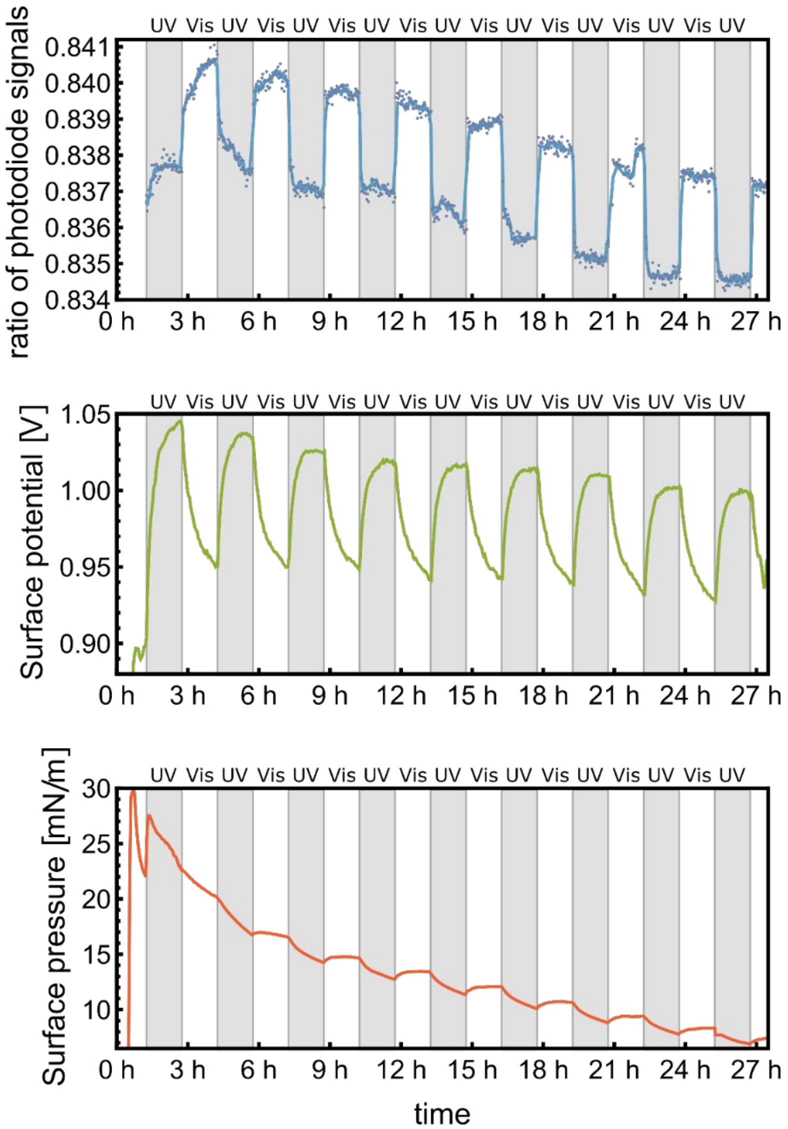

Figure S9: Changes in light absorption (blue), surface potential (green), and surface pressure (red) of a $GAzo_3$ film during irradiation with UV and blue light. The intervals with gray background indicate UV irradiation, while white background indicates irradiation with visible light. Light absorption is shown in terms of the change of the ratio of the voltage signals recorded on the two photoiodes. A brigther blue line is drawn on top of the data points for the photoiode signal: this represents the same data but filtered to reduce measurement noise.

Since a change in surface potential indicates a rotation of the molecular dipole moment, the slower change in surface potential in comparison to the change in light absorption would suggest that the molecules in the film first undergo isomerization and then slowly rotate to an energetically more favorable orientation. The mismatch between the rate of change in light absorption, surface pressure, and surface potential is in

contrast to what was reported by Maack *et al.*, where all the quantities appeared to be changing at the same rate[4].

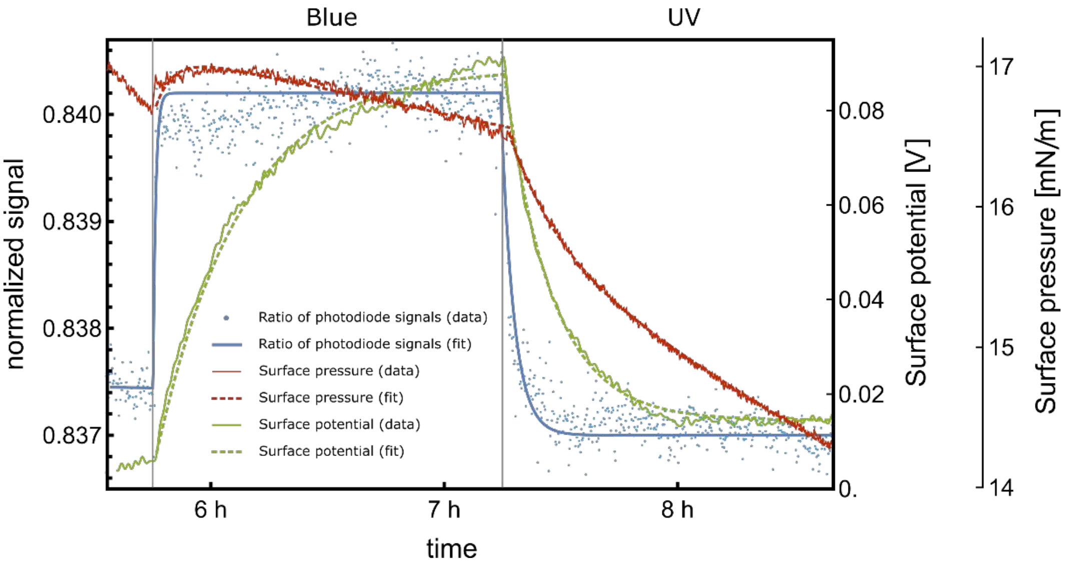

Figure S10: Surface pressure, surface potential, and light absorption of $GAzo_3$ Langmuir film during a single cycle of blue and UV irradiation. The dashed lines are exponential fits – the obtained characteristic times are written next to the lines. In the case of surface pressure, an additional linear drift term was added to the fitting function: in this case, the dotted line represents the fit without the added linear drift term.

References:

1. F. Tibiletti, M. Simonetti, K. M. Nicholas, G. Palmisano, M. Parravicini, F. Imbesi, S. Tollari, A. Penoni, *Tetrahedron* **2010**, *66* (6), 1280-1288.

2. THE CORNELL RESEARCH FOUNDATION, INC., WO2006/130161, 2006, A2, Location in patent: Page/Page column 29.

3. Ličen, M., Masiero, S. & Drevenšek-Olenik, I. Photoisomerizable Guanosine Derivative as a Probe for DNA Base-Pairing in Langmuir Monolayers. *Langmuir* **2019,** *35*, 6550–6561.

4. Maack, J., Ahuja, R. C. & Tachibana, H. Resonant and Nonresonant Investigations of Amphiphilic Azobenzene Derivatives in Solution and in Monolayers at the Air/Water Interface. *J. Phys. Chem.* **1995**, *99*, 9210–9220.
